# Inequalities in full immunization coverage: trends in low- and middle-income countries

**DOI:** 10.2471/BLT.15.162172

**Published:** 2016-08-31

**Authors:** María Clara Restrepo-Méndez, Aluísio JD Barros, Kerry LM Wong, Hope L Johnson, George Pariyo, Giovanny VA França, Fernando C Wehrmeister, Cesar G Victora

**Affiliations:** a International Center for Equity in Health, Federal University of Pelotas, Rua Marechal Deodoro 1160 – 3° Piso, Pelotas (RS), CEP:96020220, Brazil.; b Postgraduate Programme in Epidemiology, Federal University of Pelotas, Pelotas, Brazil.; c GAVI, The Vaccine Alliance, Geneva, Switzerland.; d Johns Hopkins Bloomberg School of Public Health, Baltimore, United States of America.

## Abstract

**Objective:**

To investigate disparities in full immunization coverage across and within 86 low- and middle-income countries.

**Methods:**

In May 2015, using data from the most recent Demographic and Health Surveys and Multiple Indicator Cluster Surveys, we investigated inequalities in full immunization coverage – i.e. one dose of bacille Calmette-Guérin vaccine, one dose of measles vaccine, three doses of vaccine against diphtheria, pertussis and tetanus and three doses of polio vaccine – in 86 low- or middle-income countries. We then investigated temporal trends in the level and inequality of such coverage in eight of the countries.

**Findings:**

In each of the World Health Organization’s regions, it appeared that about 56–69% of eligible children in the low- and middle-income countries had received full immunization. However, within each region, the mean recorded level of such coverage varied greatly. In the African Region, for example, it varied from 11.4% in Chad to 90.3% in Rwanda. We detected pro-rich inequality in such coverage in 45 of the 83 countries for which the relevant data were available and pro-urban inequality in 35 of the 86 study countries. Among the countries in which we investigated coverage trends, Madagascar and Mozambique appeared to have made the greatest progress in improving levels of full immunization coverage over the last two decades, particularly among the poorest quintiles of their populations.

**Conclusion:**

Most low- and middle-income countries are affected by pro-rich and pro-urban inequalities in full immunization coverage that are not apparent when only national mean values of such coverage are reported.

## Résumé

**Objectif:**

Étudier les disparités en matière de couverture vaccinale complète (disparités entre pays et au sein des pays), à partir des données obtenues pour 86 pays à revenu faible ou intermédiaire.

**Méthodes:**

En mai 2015, en utilisant des données issues des plus récentes enquêtes démographiques et sanitaires et d'enquêtes en grappes à indicateurs multiples, nous avons étudié les inégalités dans la couverture vaccinale complète (soit l'administration d'une dose du vaccin bilié de Calmette et Guérin, d'une dose de vaccin antirougeoleux, de trois doses de vaccin diphtérie-coqueluche-tétanos et de trois doses de vaccin antipoliomyélitique), dans 86 pays à revenu faible ou intermédiaire. Nous avons ensuite analysé l’évolution au fil du temps du niveau de couverture et des inégalités de cette couverture dans huit des pays étudiés.

**Résultats:**

Dans chacune des régions de l'Organisation mondiale de la Santé, près de 56 à 69% des enfants éligibles vivant dans un pays à revenu faible ou intermédiaire a bénéficié d'une couverture vaccinale complète. Néanmoins, dans chaque région, le taux de couverture moyen enregistré varie énormément. Dans la Région africaine, par exemple, ce taux est compris entre 11,4% au Tchad et 90,3% au Rwanda. Nous avons décelé une inégalité en faveur des riches dans 45 des 83 pays pour lesquels nous avons obtenu des données exploitables et une inégalité favorable aux urbains dans 35 des 86 pays étudiés. Parmi les pays pour lesquels nous avons analysé l’évolution de la couverture vaccinale au fil du temps, Madagascar et le Mozambique sont ceux qui ont fait les plus gros progrès dans l'amélioration du niveau de couverture vaccinale complète au cours des vingt dernières années, notamment auprès des quintiles les plus pauvres de leur population.

**Conclusion:**

Dans la majorité des pays à revenu faible ou intermédiaire, il existe des inégalités en faveur des riches et des urbains en matière de couverture vaccinale complète; des inégalités qui ne sont pas observables lorsque l'on considère uniquement les moyennes nationales.

## Resumen

**Objetivo:**

Investigar las disparidades en la cobertura de inmunización completa entre y dentro de 86 países con ingresos bajos y medios.

**Métodos:**

En mayo de 2015, mediante el uso de datos provenientes de las Encuestas de Demografía y Salud y las Encuestas de Conglomerados de Indicadores Múltiples más recientes, se investigaron las desigualdades en la cobertura de inmunización completa, es decir, una dosis de la vacuna del bacilo Calmette-Guérin, una dosis de la vacuna contra el sarampión, tres dosis de la vacuna contra la difteria, el tétanos y la tos ferina y tres dosis de la vacuna contra el polio, en 86 países con ingresos bajos o medios. Posteriormente, se investigaron las tendencias temporales en el nivel y la desigualdad de dicha cobertura en ocho de los países.

**Resultados:**

En todas las regiones de la Organización Mundial de la Salud, parecía que alrededor de entre un 56 y un 69% de los niños aptos en los países de ingresos bajos y medios habían recibido una inmunización completa. No obstante, dentro de cada región, el nivel medio registrado de dicha cobertura era muy diferente. En la región africana, por ejemplo, osciló de un 11,4% en Chad hasta un 90,3% en Rwanda. Se detectó una desigualdad que favorecía a los ricos en dicha cobertura en 45 de los 83 países de los cuales de disponía de información importante, y una desigualdad que favorecía a las zonas urbanas en 35 de los 86 países del estudio. Entre los países en los que se investigaron las tendencias de cobertura, Madagascar y Mozambique parecían ser los que habían progresado más a la hora de mejorar los niveles de una cobertura de inmunización completa durante las dos últimas décadas, principalmente entre la población más pobre.

**Conclusión:**

La mayoría de los países con ingresos bajos y medios se ven afectados por las desigualdades que favorecen a la población rica y a las zonas urbanas en relación con la cobertura de inmunización completa, las cuales no resultan aparentes cuando se informa únicamente de los valores nacionales medios de dicha cobertura.

## ملخص

**الغرض:**

التحقيق بشأن التفاوت في تغطية عمليات التمنيع الكاملة عبر وداخل 86 **  **من البلدان ذات الدخل المنخفض والمتوسط.

**الطريقة:**

قمنا في شهر مايو/أيار من عام **  **2015 باستخدام البيانات الواردة من أحدث المسوح الديموغرافية والصحية والمسوح العنقودية متعددة المؤشرات، وذلك للتحقيق في مسألة عدم المساواة في تغطية عمليات التمنيع الكاملة – على سبيل المثال تقديم جرعة واحدة من لقاح عُصية "كالميت غيران"، وجرعة واحدة من لقاح الحصبة، وثلاث جرعات من اللقاح ضد الدفتيريا والسعال الديكي والكزاز، وثلاث جرعات من لقاح شلل الأطفال في 86 **  **من البلدان ذات الدخل المنخفض أو المتوسط. ثم قمنا بعد ذلك بالتحقيق في النزعات الزمنية المتواجدة في مستوى مثل هذه التغطية وعدم المساواة التي تتسم بها في ثماني بلدان.

**النتائج:**

لقد بدا أن الأطفال المؤهلين في البلدان ذات الدخل المنخفض والمتوسط الذين تتراوح نسبتهم من 56 إلى 69‏% تقريبًا قد تلقوا التمنيع الكامل في كل منطقة من المناطق التابعة لمنظمة الصحة العالمية. ومع ذلك، اختلف متوسط المستوى المسجل لهذه التغطية بصورة كبيرة داخل كل منطقة. فقد اختلف مستوى التغطية على سبيل المثال في المناطق الأفريقية من ‏11.4% في تشاد إلى ‏90.3% في رواندا. واكتشفنا وجود حالات من عدم المساواة الموالية للمناطق الغنية في هذه التغطية في 45 بلدًا من إجمالي 83 بلدًا والتي توفرت البيانات ذات الصلة الخاصة بها ووجود حالات من عدم المساواة الموالية للمناطق الحضرية في 35 بلدًا من إجمالي 86 بلدًا شملتها هذه الدراسة. ومن بين البلدان التي قمنا فيها بالتحقيق بشأن نزعات التغطية، بدا أن مدغشقر وموزامبيق حققتا النسبة الأكبر من التقدم في مجال تحسين مستويات تغطية عمليات التمنيع الكاملة على مدار العقدين الماضيين، وبالأخص بين خُمس السكان الأكثر فقرًا.

**الاستنتاج:**

تتأثر معظم البلدان ذات الدخل المنخفض والمتوسط من حالات عدم المساواة الموالية للمناطق الغنية والموالية للمناطق الحضرية فيما يتعلق بتغطية عمليات التمنيع الكاملة التي لا تكون واضحة عندما يتم الإبلاغ عن قيم المتوسط الوطني فقط لهذه التغطية.

## 摘要

**目的:**

旨在调查 86 个中低收入国家之间和之内全程免疫接种率的差异。

**方法:**

2015 年 5 月，我们采用了最新人口与健康调查和多指标类集调查数据，研究了全程免疫接种率——即一剂卡介苗、一剂麻疹疫苗、三剂百日咳、白喉、破伤风疫苗和三剂脊髓灰质炎疫苗——在 86 个中低收入国家的不均衡性。 然后，我们研究了接种率水平和不均衡性在其中八个国家的变化趋势。

**结果:**

在各个世界卫生组织地区，中低收入国家 56–69% 的符合条件的儿童接受了全程免疫接种。 但是，在各个地区，所记录的平均接种率水平差别很大。 例如在非洲地区，乍得的接种率仅为 11.4%，而卢旺达的接种率高达 90.3%。 我们发现，在我们使用其相关数据的 83 个国家中，有 45 个国家的接种率体现出了贫富差距；在 86 个研究国中，有 35 个国家的接种率体现出了城市化水平的不均衡性。 在我们研究了其接种率趋势的国家中，马达加斯加和莫桑比克过去二十年间在提高全程免疫接种率水平方面取得的进步最为显著，尤其是在占其人口五分之一的最贫穷群体中。

**结论:**

大多数中低收入国家都受全程免疫接种率上贫富差距和城市化水平不均衡性的影响，而在仅报告全国接种率平均值时，这种不均衡性却不明显。

## Резюме

**Цель:**

Изучить неравномерность охвата населения полной вакцинацией в 86 странах с низким и средним уровнем дохода.

**Методы:**

В мае 2015 года на основе последних данных демографических исследований состояния здоровья населения, а также кластерных исследований с множественными показателями была изучена неравномерность охвата населения полной (т. е. включающей одну дозу вакцины БЦЖ, одну дозу противокоревой вакцины, три дозы вакцины КДС и три дозы вакцины против полиомиелита) вакцинацией в 86 странах с низким и средним уровнем дохода. Затем были исследованы тенденции во времени неравномерности охвата вакцинацией в восьми из этих стран.

**Результаты:**

Было обнаружено, что в каждом регионе Всемирной организации здравоохранения в странах с низким и средним уровнем дохода полной вакцинацией было охвачено приблизительно 56–69% отвечающих соответствующим критериям детей. Однако внутри каждого региона средний уровень вакцинации значительно различался. Например, в африканском регионе доля привитых варьировалась от 11,4% в Чаде до 90,3% в Руанде. Была обнаружена неравномерность вакцинации по признаку обеспеченности в 45 из 83 стран, для которых имелись соответствующие данные, и по признаку проживания в городе в 35 из 86 исследованных стран. Среди стран, в которых были изучены эти тенденции, Мадагаскар и Мозамбик добились наибольшего успеха в увеличении охвата населения полной вакцинацией за последние два десятилетия, в особенности среди наименее обеспеченных квинтилей населения.

**Вывод:**

В большинстве стран с низким и средним уровнем дохода существует неравномерность охвата полной вакцинацией, которая связана с уровнем обеспеченности и проживанием в городе, причем эта неравномерность не выявляется, когда учитываются только средние национальные показатели.

## Introduction

Despite the improvements made in global immunization coverage for children over the past decade,^1^^,^^2^ an estimated 21.8 million infants worldwide are still not being reached by routine immunization services.^3^ In 2013, most of the World Health Organization’s (WHO) regions reached more than 80% of their target populations with three doses of diphtheria, pertussis and tetanus (DTP) vaccine but coverage with such vaccine remained well short of the 2015 goal of 90%, particularly in the African (75%) and South-East Asia regions (77%).^2^^,^^3^ Many barriers exist to achieving good vaccination coverage, including a lack of parental education, low income, poor access to health facilities and traditional beliefs.^4^^–^^13^ As progress in this field is commonly expressed in terms of national or regional mean values, many of the underlying disparities among and within countries go unobserved or, at least, unreported. If routine immunization is to be made fast and equitable, we need multi-country studies that use the same types of stratification to document and understand the inequalities in vaccination coverage at both national and regional level.^1^^,^^3^^,^^14^^,^^15^ We also need to know the percentages of children who receive the full set of standard vaccines recommended by WHO. In India, for example, national immunization coverage has been increasing since the early 1990s but the proportion of children who, in 2006, had received all of the immunizations recommended for their age group as part of WHO’s Expanded Programme on Immunization was still under 50%.^16^ Failures or delays in the vaccination of children in high-risk groups can limit the impact of vaccine programmes on the burden of disease.^17^

The main objectives of the present analyses were: (i) to assess the proportions of children in low- or middle-income countries who receive a basic set of routine vaccinations – that is one dose of bacille Calmette-Guérin vaccine, one dose of measles vaccine, three doses of vaccine against DTP and three doses of polio vaccine – at the appropriate ages; (ii) to document between-country and within-country inequalities in such coverage – in terms of socioeconomic status and other characteristics commonly recorded in national surveys; and (iii) to assess temporal trends in such coverage and in the associated inequalities.

## Methods

In May 2015, we accessed publicly available data sets collected during the most recent Demographic and Health Survey^18^ and/or Multiple Indicator Cluster Surveys^19^ in each of the 86 low- or middle-income countries in which at least one such survey had been conducted since the year 2000 (Table 1). Our study outcome was full immunization coverage, which we defined as the proportion of children who, at any age, had received one dose of bacille Calmette-Guérin vaccine, one dose of measles vaccine, three doses of – trivalent, tetravalent or pentavalent – vaccine against DTP and three doses of polio vaccine. For the 20 study countries where measles vaccine was routinely administered at the age of 18 months, full immunization coverage was measured among children aged 18–29 months. Similarly, for the three study countries where measles vaccine was routinely administered at the age of 15 months, full immunization coverage was measured among children aged 15–26 months. Children aged 12–23 months formed the denominator group in all of the other study countries.

**Table 1:** Percentages of eligible children receiving full childhood immunization and the corresponding wealth-related inequalities in coverage, in 86 low- or middle-income countries, 2001–2012

Region, country	Year^a^	National coverage				SII			CIX	
		Overall, % (SE)^b^	In poorest quintile, % (SE)^b^	In richest quintile, % (SE)^b^		Mean percentage points (SE)	*P*		Mean (SE)	*P*
**African Region**										
Benin	2006	47.5 (1.3)	34.0 (2.3)	65.1 (2.3)		32.4 (3.5)	< 0.001		12.0 (1.3)	< 0.001
Burkina Faso	2010	81.3 (1.1)	73.3 (2.5)	83.9 (2.5)		12.9 (3.8)	0.001		2.5 (0.8)	0.001
Burundi	2010	83.1 (1.3)	78.2 (2.5)	83.9 (2.4)		7.1 (3.7)	0.053		1.2 (0.7)	0.094
Cameroon	2011	53.6 (1.7)	32.9 (3.4)	70.3 (2.7)		41.2 (4.7)	< 0.001		13.4 (1.7)	< 0.001
Central African Republic	2010	17.3 (1.5)	7.7 (1.5)	36.7 (4.2)		34.4 (4.6)	< 0.001		32.7 (3.7)	< 0.001
Chad	2004	11.4 (1.7)	1.1 (0.9)	24.0 (3.1)		25.9 (4.7)	< 0.001		34.9 (5.3)	< 0.001
Congo	2011	44.6 (2.1)	38.2 (2.6)	48.7 (5.3)		16.0 (6.2)	0.010		6.3 (2.2)	0.004
Côte d’Ivoire	2011	50.5 (2.3)	39.2 (3.4)	68.0 (4.3)		32.8 (6.0)	< 0.001		11.0 (2.0)	< 0.001
Democratic Republic of the Congo	2010	49.8 (2.2)	39.1 (3.8)	58.8 (3.7)		23.4 (5.9)	< 0.001		7.7 (2.1)	< 0.001
Ethiopia	2011	24.6 (1.9)	16.8 (2.9)	50.6 (4.7)		32.3 (5.6)	< 0.001		22.6 (3.9)	< 0.001
Gabon	2012	28.3 (2.5)	33.5 (2.9)	13.1 (3.8)		−16.8 (6.4)	0.009		−9.7 (4.0)	0.015
Gambia	2005	77.0 (1.2)	83.9 (2.1)	75.9 (3.1)		−10.0 (4.3)	0.019		−2.5 (0.9)	0.006
Ghana	2011	84.8 (1.5)	83.9 (2.7)	84.5 (3.9)		1.3 (5.1)	0.794		0.3 (1.0)	0.791
Guinea	2005	37.4 (2.3)	29.0 (4.9)	44.7 (5.2)		22.1 (7.8)	0.005		10.0 (3.6)	0.006
Guinea Bissau	2006	52.3 (2.4)	44.6 (4.2)	66.6 (4.7)		20.8 (6.9)	0.003		6.6 (2.3)	0.004
Kenya	2008	68.6 (2.0)	61.6 (4.5)	69.6 (3.5)		9.8 (6.9)	0.155		1.9 (1.7)	0.260
Lesotho	2009	62.9 (2.0)	53.3 (4.3)	73.0 (5.0)		21.3 (7.0)	0.002		5.7 (1.8)	0.002
Liberia	2007	39.1 (2.6)	23.4(4.3)	55.7 (5.5)		41.1 (6.7)	0.000		16.7 (3.4)	< 0.001
Madagascar	2008	61.7 (1.8)	41.3 (2.7)	82.8 (2.9)		51.5 (4.1)	< 0.001		14.3 (1.3)	< 0.001
Malawi	2010	81.3 (1.0)	78.5 (1.9)	82.1 (2.4)		4.3 (3.4)	0.209		0.8 (0.7)	0.235
Mali	2006	48.5 (1.9)	48.6 (3.4)	56.6 (3.6)		8.0 (5.5)	0.149		2.9 (1.9)	0.139
Mauritania	2007	35.3 (1.6)	38.1 (3.1)	24.4 (3.3)		−21.8 (5.2)	< 0.001		−9.9 (2.5)	< 0.001
Mozambique	2011	64.7 (1.8)	54.7 (3.5)	76.2 (2.8)		29.1 (4.9)	< 0.001		7.0 (1.4)	< 0.001
Namibia	2006	68.8 (1.9)	59.3 (4.1)	81.6 (5.8)		23.9 (6.7)	< 0.001		5.6 (1.7)	0.001
Niger	2012	52.5 (1.7)	35.2 (3.0)	67.1 (2.5)		33.4 (4.4)	< 0.001		11.1 (1.5)	< 0.001
Nigeria	2011	33.2 (1.3)	13.7 (1.4)	63.0 (2.9)		56.9 (3.1)	< 0.001		29.4 (1.7)	< 0.001
Rwanda	2010	90.3 (0.9)	87.1 (2.0)	95.5 (1.4)		11.0 (2.8)	< 0.001		2.1 (0.5)	< 0.001
Sao Tome and Principe	2008	76.6 (3.0)	68.1 (9.1)	81.6 (5.2)		19.1 (11.6)	0.098		4.4 (2.4)	0.067
Senegal	2010	62.9 (1.4)	56.4 (2.5)	70.0 (4.2)		13.6 (5.0)	0.007		3.7 (1.3)	0.005
Sierra Leone	2010	54.3 (2.0)	56.2 (3.7)	53.3 (4.2)		−3.1 (6.3)	0.623		−0.8 (1.9)	0.683
Swaziland	2010	85.9 (1.7)	86.5 (3.3)	78.4 (4.6)		−8.3 (5.79	0.147		−1.7 (1.0)	0.104
Togo	2010	50.4 (2.1)	36.2 (4.0)	58.9 (5.7)		25.8 (6.9)	< 0.001		8.6 (2.4)	< 0.001
Uganda	2011	52.5 (1.8)	52.1 (3.6)	55.4 (3.6)		3.3 (5.7)	0.559		1.2 (1.7)	0.479
United Republic of Tanzania	2010	75.2 (1.8)	69.1 (3.5)	84.8 (3.1)		17.5 (5.6)	0.002		4.1 (1.2)	0.001
Zambia	2007	68.4 (1.9)	72.1 (3.0)	79.7 (3.3)		5.9 (5.39	0.263		1.4 (1.3)	0.283
Zimbabwe	2010	65.9 (2.1)	57.1 (83.9)	73.4 (3.7)		21.7 (6.1)	< 0.001		5.8 (1.6)	< 0.001
**Region of the Americas**										
Belize	2006	59.2 (3.9)	56.5 (6.9)	NR^d^		1.1 (13.7)	0.934		−0.3 (3.8)	0.946
Bolivia (Plurinational State of)	2008	78.6 (1.4)	77.9 (2.7)	80.6 (3.5)		3.0 (4.8)	0.530		0.7 (1.0)	0.474
Colombia	2010	68.2 (1.1)	64.2 (2.0)	67.3 (3.4)		6.2 (3.9)	0.109		1.8 (0.9)	0.055
Costa Rica	2011	90.1 (2.7)	86.2 (6.5)	90.9 (6.7)^c^		−0.6 (11.1)	0.958		0.0 (1.9)	0.997
Cuba	2010	78.9 (3.7)	NA	NA		NA	NA		NA	NA
Dominican Republic	2007	58.2 (1.6)	49.1 (2.3)	75.6 (4.3)		24.2 (5.4)	< 0.001		7.6 (1.5)	< 0.001
Guyana	2009	63.4 (2.9)	59.9 (4.7)	57.9 (6.6)		1.5 (9.9)	0.882		0.0 (2.3)	0.986
Haiti	2012	45.8 (2.0)	42.9 (4.3)	42.6 (5.0)		−1.0 (7.0)	0.881		−0.3 (2.5)	0.904
Honduras	2011	85.1 (1.1)	87.2 (1.4)	87.8 (2.4)		−1.7 (3.7)	0.646		−0.5 (0.7)	0.473
Jamaica	2005	74.3 (2.8)	NA	NA		NA	NA		NA	NA
Nicaragua	2001	72.0 (1.6)	64.0 (3.1)	71.5 (4.9)		9.4 (6.2)	0.131		2.4 (1.4)	0.079
Peru	2009	52.6 (1.6)	45.7 (2.7)	52.6 (5.7)		10.2 (6.1)	0.093		3.5 (1.9)	0.060
**South-East Asia Region**										
Bangladesh	2011	86.0 (1.2)	76.8 (3.0)	93.6 (1.7)		19.3 (4.2)	< 0.001		3.6 (0.8)	< 0.001
India	2005	43.7 (0.9)	24.4 (1.4)	71.0 (1.5)		53.8 (2.1)	< 0.001		21.3 (1.0)	< 0.001
Indonesia	2012	65.7 (1.3)	47.4 (2.4)	80.2 (2.2)		35.9 (3.6)	< 0.001		9.6 (1.0)	< 0.001
Maldives	2009	92.9 (1.2)	94.7 (1.8)	92.2 (3.5)		−5.8 (4.4)	0.184		−1.0 (0.7)	0.193
Nepal	2011	87.1 (2.1)	85.0 (2.8)	95.7 (2.2)		12.0 (4.5)	0.008		2.3 (0.9)	0.012
Thailand	2005	89.7 (1.1)	91.7 (1.7)	86.0 (3.0)		−7.6 (3.5)	0.032		−1.2 (0.6)	0.065
Timor-Leste	2009	52.6 (1.8)	43.2 (3.1)	45.2 (3.9)		9.6 (5.8)	0.097		3.0 (1.8)	0.106
**European Region**										
Albania	2008	95.1 (1.7)	100.0 (0.0)	96.9 (3.0)^c^		−4.8 (4.6)	0.300		−0.4 (0.7)	0.560
Armenia	2010	91.5 (2.1)	88.3 (6.7)	90.2 (5.2)^c^		−2.0 (8.4)	0.811		−0.9 (1.5)	0.577
Azerbaijan	2006	59.4 (3.1)	47.6 (5.5)	74.0 (8.3)		27.4 (10.2)	0.007		7.8 (2.9)	0.006
Belarus	2005	97.6 (0.6)	100.0 (0.0)	97.5 (1.3)		−1.7 (1.8)	0.334		−0.3 (0.3)	0.266
Bosnia and Herzegovina	2011	85.0 (1.9)	87.6 (3.4)	77.8 (5.4)		−8.6 (7.2)	0.229		−1.4 (1.3)	0.311
Georgia	2005	19.5 (2.5)	17.0 (5.2)	17.8 (5.4)		1.1 (8.8)	0.900		1.3 (7.8)	0.867
Kazakhstan	2010	84.3 (1.6)	84.0 (3.8)	84.5 (2.8)		−1.6 (5.8)	0.782		−0.4 (1.1)	0.751
Kyrgyzstan	2005	21.6 (3.6)	15.2 (4.3)	33.9 (5.5)		17.9 (8.7)	0.040		−14.4 (7.5)	0.053
Montenegro	2005	56.8 (4.3)	46.1 (9.2)^c^	46.5 (9.7)^c^		9.7 (14.3)	0.497		1.8 (4.1)	0.672
Republic of the Moldova	2005	42.5 (3.0)	25.2 (6.6)^c^	50.8 (4.9)		28.8 (8.7)	0.001		11.0 (3.7)	0.003
Serbia	2005	47.9 (2.6)	40.2 (5.1)	49.9 (5.6)		19.4 (8.1)	0.016		7.3 (2.9)	0.011
Tajikistan	2012	88.9 (1.3)	88.7 (3.7)	86.7 (2.4)		−5.1 (4.8)	0.291		−1.0 (0.9)	0.291
The former Yugoslav Republic of Macedonia	2011	92.6 (1.9)	91.7 (4.3)	91.9 (4.4)		−0.9 (7.1)	0.905		−0.3 (1.2)	0.825
Turkey	2003	54.4 (2.4)	29.3 (3.9)	70.6 (4.6)		51.6 (6.0)	< 0.001		16.7 (2.2)	< 0.001
Uzbekistan	2006	86.9 (1.6)	90.1 (2.6)	80.0 (3.6)		−12.6 (4.9)	0.011		−2.5 (0.9)	0.007
**Eastern Mediterranean Region**										
Afghanistan	2010	17.1 (1.6)	12.9 (3.1)	22.6 (2.4)		12.0 (5.3)	0.022		12.0 (5.5)	0.030
Djibouti	2006	39.0 (3.0)	NA	NA		NA	NA		NA	NA
Egypt	2008	91.8 (0.7)	89.6 (1.5)	94.4 (1.4)		6.5 (2.4)	0.006		1.2 (0.4)	0.003
Iraq	2011	63.0 (1.0)	47.4 (1.7)	73.2 (2.7)		32.8 (3.2)	< 0.001		8.9 (0.9)	< 0.001
Jordan	2012	93.1 (1.2)	89.7 (2.3)	91.1 (5.3)		3.5 (5.5)	0.526		0.5 (1.0)	0.600
Morocco	2003	89.2 (1.1)	81.0 (3.1)	97.4 (1.2)		21.8 (4.2)	< 0.001		3.9 (0.7)	< 0.001
Pakistan	2012	53.9 (2.2)	23.4 (4.3)	75.4 (3.3)		55.1 (5.4)	< 0.001		18.3 (2.2)	< 0.001
Somalia	2006	11.6 (1.6)	5.4 (2.5)	21.7 (4.0)		21.1 (5.6)	< 0.001		29.0 (6.5)	< 0.001
Syrian Arab Republic	2006	65.9 (1.4)	49.9 (3.1)	76.2 (2.5)		27.0 (4.4)	< 0.001		7.0 (1.1)	< 0.001
Yemen	2006	30.6 (2.4)	14.0 (3.3)	57.8 (5.4)		50.1 (6.2)	< 0.001		26.7 (3.7)	< 0.001
**Western Pacific Region**										
Cambodia	2010	78.8 (1.5)	65.3 (3.0)	88.2 (2.2)		28.3 (4.5)	< 0.001		6.4 (1.0)	0.000
Lao People's Democratic Republic	2011	43.7 (1.6)	29.6 (2.7)	61.7 (3.4)		39.9 (4.7)	< 0.001		15.9 (2.0)	0.000
Mongolia	2010	78.5 (1.9)	78.2 (3.0)	78.0 (4.5)		1.5 (5.7)	0.794		0.3 (1.2)	0.825
Philippines	2008	79.5 (1.3)	63.6 (2.9)	87.1 (2.7)		30.5 (4.4)	< 0.001		6.2 (0.9)	0.000
Vanuatu	2007	38.0 (3.3)	22.5 (5.9)	45.3 (6.4)		20.0 (10.5)	0.056		10.0 (4.5)	0.027
Viet Nam	2010	60.7 (2.4)	46.3 (5.4)	71.8 (4.2)		27.1 (8.1)	0.001		7.4 (2.4)	0.002

CIX: concentration index; NA: not available; NR: not reported; SE: standard error; SII: slope index of inequality.

^a^ Year in which the Demographic and Health Survey^18^ or Multiple Indicator Cluster Survey^19^ providing the coverage data was conducted.

^b^ The mean proportion of eligible children included in the survey who, at any age, had received one dose of bacille Calmette-Guérin vaccine, one dose of measles vaccine, three doses of – trivalent, tetravalent or pentavalent – vaccine against diphtheria, pertussis and tetanus and three doses of polio vaccine.

^c^ Value based on a small sample of 25–50 children.

^d^ Not reported or included in our analysis because sample was less than 25 children.

### Inequalities in coverage

We investigated inequalities in full immunization coverage that related to three characteristics recorded in all or most of the surveys that had provided the data that we used: socioeconomic status, urban/rural residence and sex of the child. The data for three study countries – Cuba, Djibouti and Jamaica – could not be used to estimate the wealth index that we used as a measure of socioeconomic status. We calculated a mean full immunization coverage for the study countries in each WHO region.

In each of the surveys we used as a data source, urban or rural residence had been defined by the local census bureaux and the study households had been categorized into five asset-based wealth quintiles. The quintiles had been derived, using principal component analyses, from variables representing household goods, materials used for housing construction and available infrastructure such as types of water access and sanitation facilities.^20^

To summarize any wealth-related inequalities in full immunization coverage, we calculated four indicators.^21^ Two of these were based on simple comparisons of the coverage recorded for the lowest wealth quintile and that recorded for the highest wealth quintile: (i) the difference, in percentage points, between the two values; and (ii) the ratio between the two values. We also calculated two indicators of inequality that take the whole distribution of wealth into account: (i) the slope index of inequality – which uses a logistic regression model to express the absolute difference in coverage, in percentage points, between the extremes of the wealth distribution;^22^ and (ii) a concentration index^23^ that is similar in concept to the Gini index for income distribution. The concentration index was expressed on a scale from −100 to +100, with full equality indicated by a value of zero. Both summary indicators tended to be positive, indicating that full immunization coverage was higher for the rich than for the poor. We calculated standard errors for each summary indicator and corresponding *P*-values for the probability that there was no inequality. Our absolute measures of inequality – i.e. the difference in coverage between the lowest and highest wealth quintiles and the slope index of inequality – give an idea of the effort that will be needed to close the gap. Our two relative measures – i.e. the ratio between the coverage for the lowest and highest wealth quintiles and the concentration index – give an idea of the degree of disparity.

We made similar comparisons of the coverage recorded for urban children and that recorded for rural children – again, the difference, in percentage points, between the two values and the ratio between the two values. We evaluated the statistical significance of the difference, assuming a binomial distribution. In a similar fashion, we investigated inequalities in coverage according to the sex of the child.

Standard errors, expressed in percentage points, were calculated for all of the coverage estimates. Like the tests for statistical significance, these took into account the sample weights and clustering. When the unweighted number of children in a specific subgroup – e.g. a wealth quintile – was less than 25, we ignored the results for that subgroup.

### Temporal trends in inequalities

Time-trend analyses were conducted for a subset of eight low-income study countries that had previously been identified as having the greatest within-country disparity in vaccine coverage: Central African Republic, Chad, India, Madagascar, Mozambique, Nigeria, Pakistan and Viet Nam.^24^ We investigated the temporal trends in routine immunization coverage , for children aged no more than 23 months, by both wealth quintile and urban/rural residence. Whenever possible, pre-2000, 2000 – or close to 2000 – and post-2000 records were included (available from the corresponding author) to cover periods before Gavi was launched, when Gavi was launched and when Gavi’s main strategies had been implemented, respectively.

Variance-weighted least squares regression was used, with survey as the independent variable, to test the statistical significance of the observed temporal trends, taking into account the clustered nature of the survey samples.

## Results

### Detected inequalities

#### Wealth

Table 1 shows national levels of full immunization coverage for all 86 countries that we assessed. For the 83 study countries for which the relevant data were available, it also summarizes the full immunization coverage for the lowest and highest wealth quintiles and the corresponding summary indicators of inequality according to wealth. Five countries –Afghanistan, Central African Republic, Chad, Georgia and Somalia – showed national levels of full immunization coverage that were below 20%. Nine countries –Albania, Armenia, Belarus, Costa Rica, Egypt, Jordan, Maldives, Rwanda and The former Yugoslav Republic of Macedonia – showed corresponding coverage above 90%. Lesotho and Senegal, each with a full immunization coverage of 63%, represented the 50th percentile of the country ranking. In most of the study countries, the increase in coverage with wealth was monotonic, that is, coverage in the lowest wealth quintile was lower than that in the second-lowest, coverage in the second-lowest quintile was lower than that in the third-lowest and so on.

Of the 83 study countries for which the relevant data on wealth were available, 65 each gave a positive slope index of inequality that indicated the existence of a pro-rich inequality in coverage (Table 1). For 45 of the countries with a positive slope index of inequality, that index was significantly different from zero. Although 18 countries had negative slope indices, indicating a pro-poor inequality in coverage, only five of the 18 negative slope indices were significantly different from zero. The corresponding results for the concentration index were very similar: we recorded 64 positive and 19 negative concentration indices, of which 45 and four, respectively, were significantly different from zero.

In terms of the slope indices of inequality, Nigeria showed the greatest pro-rich inequality in full immunization coverage, followed by Pakistan, India, Turkey, Madagascar, Yemen, Cameroon and Liberia. The corresponding patterns for the concentration indices were similar. Seven countries – in descending order of pro-rich inequality, Chad, Central African Republic, Nigeria, Somalia, Yemen, Ethiopia and India – gave concentration indices above 20 (Table 1). In terms of one or both of our summary indicators, only four of our study countries showed distinctively pro-poor inequalities in their full immunization coverage: Gabon, Gambia, Mauritania and Uzbekistan. However, Gabon and Mauritania had relatively low national levels of coverage.

Countries that appeared similar in terms of their national values for full immunization coverage could show very different degrees of inequality. For example, Côte d’Ivoire and Mali had national values of about 50% but very different slope indices of inequality – of 8 and 33 percentage points, respectively – and very different concentration indices – of 3 and 11, respectively. Likewise, the Plurinational State of Bolivia and Philippines had national values of about 79% but very different slope indices – of 3 and 30 percentage points, respectively – and very different concentration indices – of 0.7 and 6, respectively.

**Fig. 1:**
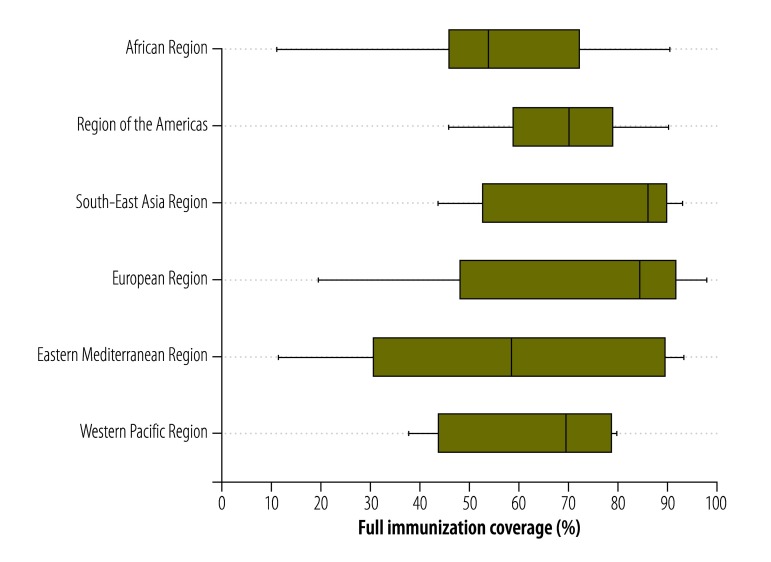
Full childhood immunization coverage in low- or middle-income countries by World Health Organization region, 2001–2012

Notes: Full coverage indicates the proportion of eligible children, included in national surveys, conducted between 2001 and 2012, who, at any age, had received one dose of bacille Calmette-Guérin vaccine, one dose of measles vaccine, three doses of – trivalent, tetravalent or pentavalent – vaccine against diphtheria, pertussis and tetanus and three doses of polio vaccine. The data are presented as box plots. The left and right side of each box indicate the 25th and 75th percentiles: the line dissecting the box is the median value. The whiskers indicate the range of values.

**Fig. 2:**
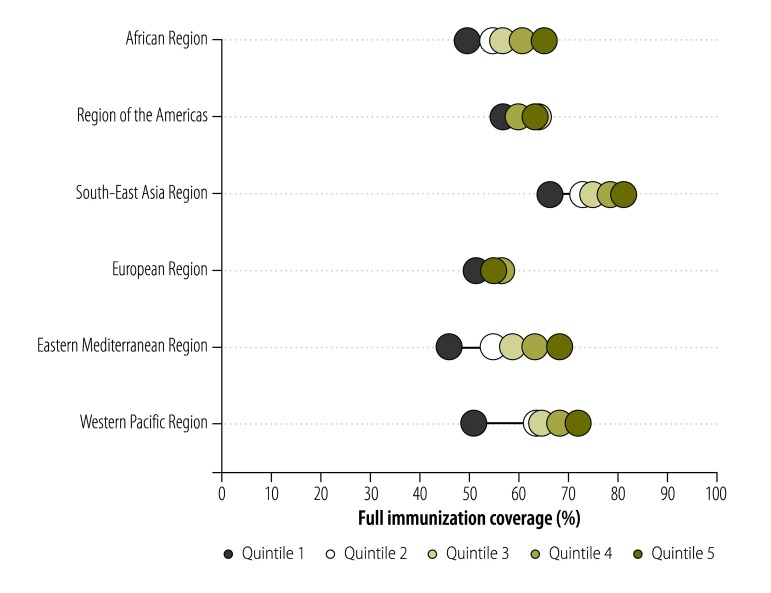
Mean full childhood immunization coverage in low- or middle-income countries split by wealth quintile, by World Health Organization region, 2001–2012

Notes: Full coverage indicates the proportion of eligible children, included in national surveys, conducted between 2001 and 2012, who, at any age, had received one dose of bacille Calmette-Guérin vaccine, one dose of measles vaccine, three doses of – trivalent, tetravalent or pentavalent – vaccine against diphtheria, pertussis and tetanus and three doses of polio vaccine. The mean values shown were not weighted according to the sizes of the national populations. The plots show coverage recorded in each wealth quintile, from the poorest – i.e. quintile 1 – to the richest – i.e. quintile 5.

Table 2 (available at: http://www.who.int/bulletin/volumes/94/11/15-162172) shows the mean values for full immunization coverage in the low- and middle-income countries we investigated in each WHO Region, which varied from 55.5% in the Eastern Mediterranean Region to 68.9% in the Region of the Americas. Globally, according to the most recent survey data available in May 2015, just over 60% of all eligible children in low- and middle-income countries had received full immunization. There was wide variation in the level of full immunization coverage within a given Region (Fig. 1). For example, in the African Region, the mean level of full immunization coverage varied from just 11.4% in Chad to 90.3% in Rwanda. Fig. 2 shows that on average, the wealth inequalities in full immunization coverage were less marked in the low- and middle-income countries in the Region of the Americas and the European Region than in such countries in other regions. The Eastern Mediterranean Region not only presented the highest absolute and relative wealth-related inequalities in such coverage but also the lowest mean level of such coverage.

**Table 2:** Full immunization coverage, and levels of sex-related, urban/rural and wealth-related inequalities in such coverage, in low- and middle-income countries by World Health Organization region, 2001–2012

Region	Mean coverage, %^a^	Mean sex-related inequality			Mean urban/rural inequality			Mean wealth-related inequality			
		Male coverage – female coverage, percentage points	Male coverage/female coverage		Urban coverage – rural coverage, percentage points	Urban coverage/rural coverage		SII, percentage points	CIX	Q5 coverage – Q1 coverage, percentage points	Q5 coverage/Q1 coverage
African Region	56.7	−0.2	1.0		7.0	1.2		17.8	7.3	15.4	2.1
Region of the Americas	68.9	1.1	1.0		−1.0	1.0		5.2	1.5	4.0	1.1
South-East Asia Region	74.0	2.5	1.0		2.6	1.1		16.7	5.4	14.4	1.4
European Region	68.2	1.9	1.1		3.4	1.1		7.9	1.6	6.5	1.3
Eastern Mediterranean Region	55.5	0.3	1.1		11.2	1.6		25.5	11.9	21.8	2.2
Western Pacific Region	63.2	−3.3	0.9		9.0	1.2		24.6	7.7	21.1	1.6

CIX: concentration index; Q1; poorest quintile; Q5: richest quintile; SII: slope index of inequality

^a^ The mean proportion of eligible children included in national surveys who, at any age, had received one dose of bacille Calmette-Guérin vaccine, one dose of measles vaccine, three doses of – trivalent, tetravalent or pentavalent – vaccine against diphtheria, pertussis and tetanus and three doses of polio vaccine. Mean values were not weighted by the sizes of the national populations.

#### Residence

Table 3 summarizes mean levels of full immunization coverage, split according to urban/rural residence, in our 86 study countries. Although 28 countries had higher levels of coverage in their rural areas than in their urban areas, the differences were generally very small and only those for Mauritania, Swaziland and Uzbekistan were statistically significant. The remaining 58 countries had higher levels of coverage in their urban areas than in their rural areas and the differences for 35 of these countries achieved statistical significance. In Ethiopia, which showed the greatest absolute pro-urban inequality, the mean level of full immunization coverage in urban areas was 28 percentage points higher than that in rural areas. Côte d’Ivoire, Madagascar, Nigeria, Turkey and Yemen also showed pro-urban differences of at least 20 percentage points. In contrast, Mauritania, Swaziland and Uzbekistan showed pro-rural differences of at least 10 percentage points. In terms of full immunization coverage, no other countries showed a statistically significant advantage for rural children.

**Table 3:** Sex-related and urban/rural inequalities in full childhood immunization coverage in 86 low- or middle-income countries, 2001–2012

Region, country	Year^a^	Area of residence, mean coverage (SE)**^b^**			Sex of child, mean coverage (SE)**^b^**	
		Urban	Rural		Male	Female
**African Region**						
Benin	2006	55.0 (2.1)	43.4 (1.6)		46.6 (1.6)	48.4 (1.6)
Burkina Faso	2010	81.7 (2.3)	81.3 (1.3)		82.1 (1.3)	80.6 (1.4)
Burundi	2010	84.0 (2.6)	83.0 (1.4)		83.6 (1.5)	82.5 (1.7)
Central African Republic	2010	63.2 (2.0)	46.7 (2.4)		17.6 (1.6)	16.5 (1.8)
Cameroon	2011	29.2 (2.9)	11.0 (1.3)		52.3 (2.2)	54.8 (1.8)
Chad	2004	21.3 (2.9)	9.0 (1.9)		11.0 (1.9)	11.8 (1.7)
Congo	2011	47.8 (3.1)	39.6 (2.4)		47.4 (2.7)	41.8 (2.6)
Côte d’Ivoire	2011	63.0 (3.1)	42.2 (2.7)		53.1 (3.0)	48.1 (2.6)
Democratic Republic of the Congo	2010	54.6 (2.6)	48.1 (2.8)		50.7 (2.3)	48.7 (2.4)
Ethiopia	2011	48.2 (5.2)	20.6 (1.9)		23.1 (2.1)	26.1 (2.2)
Gabon	2012	27.5 (2.9)	32.3 (3.3)		30.3 (3.3)	32.3 (3.0)
Gambia	2005	74.4 (2.0)	78.3 (1.5)		75.5 (1.7)	80.0 (1.5)
Ghana	2011	82.3 (2.5)	86.7 (1.8)		83.5 (2.4)	86.1 (1.8)
Guinea	2005	40.3 (4.7)	36.6 (2.6)		38.8 (2.7)	35.9 (2.7)
Guinea Bissau	2006	59.4 (3.6)	49.3 (3.0)		49.8 (2.7)	49.9 (2.7)
Kenya	2008	63.2 (3.4)	70.2 (2.3)		71.3 (3.1)	66.0 (2.8)
Lesotho	2009	71.5 (4.6)	60.4 (2.3)		59.1 (3.0)	66.4 (2.6)
Liberia	2007	52.5 (3.3)	32.7 (3.4)		36.1 (2.7)	42.6 (3.3)
Madagascar	2008	81.1 (2.5)	59.4 (2.0)		61.4 (2.0)	61.9 (2.0)
Malawi	2010	76.2 (3.5)	82.2 (1.0)		81.4 (1.2)	81.1 (1.4)
Mali	2006	53.7 (3.6)	46.4 (2.3)		51.8 (2.0)	45.2 (2.3)
Mauritania	2007	25.3 (2.3)	42.4 (2.0)		35.1 (2.1)	37.5 (2.2)
Mozambique	2011	75.4 (2.1)	60.7 (2.2)		63.8 (2.1)	65.5 (2.1)
Namibia	2006	71.5 (3.2)	67.0 (2.3)		66.9 (2.3)	70.8 (2.6)
Niger	2012	68.7 (2.8)	49.7 (2.0)		51.9 (2.1)	53.0 (2.0)
Nigeria	2011	49.6 (2.8)	25.3 (1.2)		33.2 (1.4)	32.7 (1.6)
Rwanda	2010	94.8 (2.1)	89.8 (1.0)		90.2 (1.0)	90.4 (1.0)
Sao Tome and Principe	2008	76.1 (5.0)	77.0 (3.6)		78.1 (4.3)	74.8 (4.3)
Senegal	2010	63.1 (2.6)	62.7 (1.7)		62.9 (1.9)	62.8 (1.8)
Sierra Leone	2010	51.4 (3.1)	55.5 (2.6)		53.3 (2.6)	49.2 (2.5)
Swaziland	2010	77.3 (4.3)	87.8 (1.8)		85.3 (2.2)	86.4 (2.4)
Togo	2010	57.1 (3.8)	47.5 (2.6)		48.8 (2.8)	51.7 (2.8)
Uganda	2011	61.5 (3.5)	51.1 (2.0)		52.5 (2.5)	52.6 (2.3)
United Republic of Tanzania	2010	85.6 (2.4)	72.6 (2.0)		75.8 (2.1)	74.5 (2.4)
Zambia	2007	72.2 (2.5)	67.0 (2.4)		69.2 (2.3)	67.6 (2.4)
Zimbabwe	2010	70.3 (3.9)	64.1 (2.4)		64.6 (2.6)	67.2 (2.6)
**Region of the Americas**						
Belize	2006	59.6 (6.0)	58.7 (4.8)		68.8 (5.2)	52.3 (5.9)
Bolivia (Plurinational State of)	2008	78.1 (2.0)	79.2 (2.0)		78.5 (1.8)	78.8 (1.9)
Colombia	2010	68.8 (1.3)	66.5 (2.0)		70.1 (1.4)	66.1 (1.6)
Costa Rica	2011	89.5 (4.3)	90.9 (2.2)		86.8 (3.6)	89.5 (4.1)
Cuba	2010	77.2 (4.3)	83.9 (6.0)		77.4 (5.3)	80.4 (3.5)
Dominican Republic	2007	57.0 (2.1)	60.9 (2.1)		57.6 (2.2)	58.9 (2.5)
Guyana	2009	60.2 (6.6)	64.3 (3.2)		62.4 (3.5)	64.4 (4.0)
Haiti	2012	44.5 (2.9)	46.5 (2.6)		47.2 (2.6)	44.3 (2.6)
Honduras	2011	82.7 (1.7)	87.1 (1.5)		85.5 (1.4)	84.5 (1.5)
Jamaica	2005	73.8 (4.2)	74.9 (3.5)		75.0 (3.8)	78.4 (3.8)
Nicaragua	2001	74.4 (2.3)	69.8 (2.3)		73.3 (2.0)	70.7 (2.2)
Peru	2009	54.4 (2.1)	49.0 (2.4)		52.2 (2.2)	53.0 (2.4)
**South-East Asia Region**						
Bangladesh	2011	86.6 (2.1)	85.8 (1.5)		87.3 (1.6)	84.7 (1.6)
India	2005	57.7 (1.5)	38.7 (1.0)		45.5 (1.0)	41.6 (1.0)
Indonesia	2012	69.6 (1.8)	62.0 (1.9)		66.2 (1.7)	65.2 (1.7)
Maldives	2009	91.4 (2.9)	93.5 (1.1)		93.4 (1.6)	92.3 (1.6)
Nepal	2011	90.0 (2.3)	86.8 (2.3)		88.4 (2.3)	85.7 (2.3)
Thailand	2005	87.0 (2.2)	90.7 (1.2)		92.3 (1.1)	89.6 (1.5)
Timor-Leste	2009	47.7 (3.2)	54.1 (2.1)		54.3 (2.2)	50.8 (2.1)
**European Region**						
Albania	2008	97.1 (2.0)	93.9 (2.5)		95.2 (1.1)	95.1 (2.2)
Armenia	2010	90.9 (2.7)	92.3 (3.4)		91.3 (2.6)	91.7 (3.0)
Azerbaijan	2006	67.6 (4.5)	51.7 (3.9)		55.5 (3.9)	44.0 (4.5)
Belarus	2005	97.5 (0.7)	98.0 (1.2)		98.5 (0.7)	96.8 (0.9)
Bosnia and Herzegovina	2011	85.6 (2.5)	84.8 (2.6)		87.1 (2.1)	82.8 (2.9)
Georgia	2005	19.2 (3.8)	19.8 (3.3)		11.0 (2.6)	9.5 (2.8)
Kazakhstan	2010	82.1 (2.0)	86.2 (2.4)		84.7 (2.2)	83.0 (1.9)
Kyrgyzstan	2005	31.2 (3.8)	15.5 (5.2)		35.0 (5.2)	29.8 (5.5)
Montenegro	2005	56.5 (5.7)	57.4 (6.1)		58.0 (5.5)	58.7 (5.5)
Republic of the Moldova	2005	44.5 (3.9)	41.3 (4.3)		87.8 (2.8)	87.1 (2.8)
Serbia	2005	54.1 (3.6)	40.7 (3.5)		49.2 (3.3)	50.1 (3.5)
Tajikistan	2012	87.7 (2.0)	89.2 (1.6)		89.3 (1.6)	88.4 (1.7)
The former Yugoslav Republic of Macedonia	2011	89.7 (3.4)	95.3 (1.9)		90.2 (3.1)	93.2 (2.6)
Turkey	2003	62.9 (2.8)	36.9 (3.8)		57.7 (2.9)	50.9 (2.8)
Uzbekistan	2006	78.1 (3.4)	90.4 (1.7)		86.9 (1.9)	87.9 (2.0)
**Eastern Mediterranean Region**						
Afghanistan	2010	20.5 (2.3)	16.4 (1.9)		35.9 (2.1)	33.6 (2.3)
Djibouti	2006	39.6 (3.1)	21.1 (6.9)^c^		39.2 (4.2)	39.5 (4.4)
Egypt	2008	93.7 (0.9)	90.6 (0.9)		91.0 (0.9)	92.6 (0.9)
Iraq	2011	68.9 (1.2)	50.6 (1.6)		63.3 (1.2)	61.9 (1.2)
Jordan	2012	93.2 (1.5)	92.8 (1.5)		92.7 (1.9)	93.5 (1.4)
Morocco	2003	93.5 (1.1)	84.2 (1.9)		87.0 (1.4)	91.2 (1.3)
Pakistan	2012	65.9 (2.5)	48.6 (2.9)		56.2 (2.5)	51.6 (2.4)
Somalia	2006	21.5 (3.4)	5.6 (1.2)		13.5 (2.2)	8.0 (1.4)
Syrian Arab Republic	2006	67.7 (1.8)	64.1 (2.0)		67.0 (1.7)	69.8 (1.7)
Yemen	2006	45.6 (4.0)	24.4 (2.7)		37.8 (3.1)	39.0 (3.4)
**Western Pacific Region**						
Cambodia	2010	85.5 (2.1)	77.4 (1.8)		77.1 (2.1)	80.5 (1.9)
Lao People's Democratic Republic	2011	54.9 (3.2)	40.2 (1.9)		41.9 (1.8)	45.1 (2.0)
Mongolia	2010	80.5 (2.7)	75.4 (2.5)		76.5 (2.5)	79.7 (2.6)
Philippines	2008	82.3 (1.9)	76.8 (1.8)		80.5 (1.6)	78.5 (1.8)
Vanuatu	2007	43.9 (5.1)	36.8 (3.9)		39.7 (3.9)	44.3 (4.9)
Viet Nam	2010	70.1 (3.3)	56.4 (3.1)		57.8 (3.1)	65.1 (3.0)

SE: standard error.

^a^ Year in which the Demographic and Health Survey^18^ or Multiple Indicator Cluster Survey^19^ providing the coverage data was conducted.

^b^ The mean proportion of eligible children included in the survey who, at any age, had received one dose of bacille Calmette-Guérin vaccine, one dose of measles vaccine, three doses of – trivalent, tetravalent or pentavalent – vaccine against diphtheria, pertussis and tetanus and three doses of polio vaccine.

^c^ Value based on a small sample of 25–50 children.

**Fig. 3:**
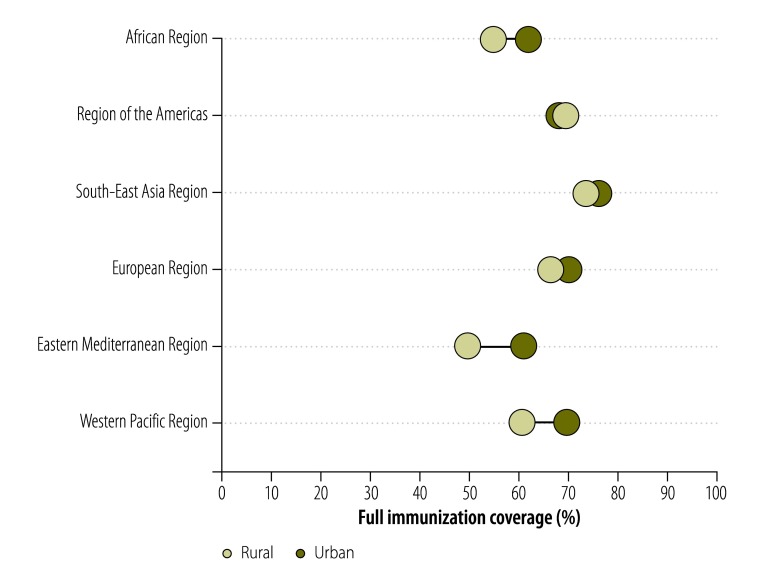
Mean full childhood immunization coverage in low- or middle-income countries split by urban or rural residence, by World Health Organization region, 2001–2012

Notes: Full coverage indicates the proportion of eligible children, included in national surveys, conducted between 2001 and 2012, who, at any age, had received one dose of bacille Calmette-Guérin vaccine, one dose of measles vaccine, three doses of – trivalent, tetravalent or pentavalent – vaccine against diphtheria, pertussis and tetanus and three doses of polio vaccine. The mean values shown were not weighted according to the sizes of the national populations.

In terms of the effects of urban/rural residence on full immunization coverage (Table 2, available at: http://www.who.int/bulletin/volumes/94/11/15-162172, and Fig. 3), the Region of the Americas and the European Region appeared more equitable than other Regions. The Eastern Mediterranean Region showed the largest pro-urban inequalities, where mean levels of full immunization coverage were about 60% higher among urban children than among their rural counterparts.

#### Sex of child

**Fig. 4:**
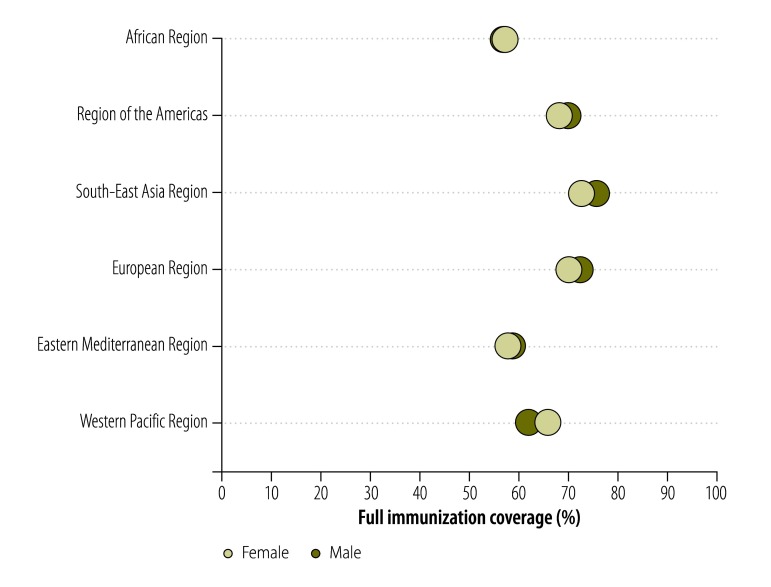
Mean full childhood immunization coverage in low- or middle-income countries split by sex of the child, by World Health Organization region, 2001–2012

Notes: Full coverage indicates the proportion of eligible children, included in national surveys, conducted between 2001 and 2012, who, at any age, had received one dose of bacille Calmette-Guérin vaccine, one dose of measles vaccine, three doses of – trivalent, tetravalent or pentavalent – vaccine against diphtheria, pertussis and tetanus and three doses of polio vaccine. The mean values shown were not weighted according to the sizes of the national populations.

Although most of our study countries showed higher levels of full immunization coverage among boys than girls (Table 3), such sex-related differences were of less than three percentage points in each of 59 countries and only achieved statistical significance in Azerbaijan, Belize, India, Mali and Somalia. The absolute levels of sex-related inequality were relatively high in the South-East Asia and Western Pacific Regions. In the Western Pacific Region, the mean level of full immunization coverage was 10% lower among boys than among girls (Fig. 4).

### Temporal trends

**Fig. 5:**
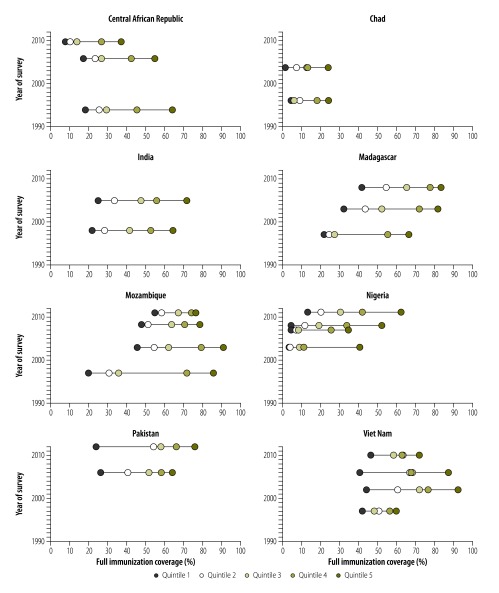
Temporal trends in wealth-related inequalities in the full childhood immunization coverage in eight countries, 1994–2012

Notes: Full coverage indicates the proportion of eligible children, included in national surveys, conducted between 1994 and 2012, who, at any age, had received one dose of bacille Calmette-Guérin vaccine, one dose of measles vaccine, three doses of – trivalent, tetravalent or pentavalent – vaccine against diphtheria, pertussis and tetanus and three doses of polio vaccine. The plots show coverage recorded in each wealth quintile, from the poorest – i.e. quintile 1 – to the richest – i.e. quintile 5.

In all eight countries included in our investigation of temporal trends, there was evidence of pro-rich inequality in full immunization coverage at all of the time-points we investigated (Fig. 5; available at: http://www.who.int/bulletin/volumes/94/11/15-162172). Thus, for any country at any time, the poorest wealth quintile had the lowest coverage. However, over the period we investigated, there were substantial differences between the countries in terms of the national trend in full immunization coverage, the degree of inequality in such coverage and the temporal changes in inequality associated with wealth quintile or urban/rural place of residence (available from the corresponding author).

In the Central African Republic, for example, there was a major decline in the national level of full immunization coverage over our study period. The level of absolute pro-rich inequality declined – since the absolute reduction in coverage was most marked among the richest quintile – but the level of relative pro-rich inequality increased. In Chad, however, the national level of coverage remained low and stable over our study period and wealth-related and urban/rural inequalities remained largely unchanged. In India, the national level of coverage increased but, as in Chad, wealth-related and urban/rural inequalities remained fairly stable. The results for Madagascar and Mozambique, which both showed increasing national levels of coverage over time, were more encouraging. The relative pro-rich inequality observed in Madagascar also decreased over time, although the absolute pro-rich inequality and all measures of urban/rural inequality did not decline. In Mozambique, much of the increase seen in the national level of coverage was linked to increasing coverage in the poorest quintile of the population. Although the country’s pro-rich and pro-urban inequalities decreased over time, the decrease observed in the pro-rich inequality was partly attributable to a decrease in coverage among the children from the richest quintile. In Nigeria, increases in the level of full immunization coverage in some areas had little impact on the overall national level, which remained relatively low. When we compared the most recent data we investigated for each of the eight countries, Nigeria showed the largest absolute pro-rich inequality as well as a large level of pro-urban inequality. Although Nigeria’s relative pro-rich inequality appeared to have decreased over our study period, this was only the result of mean coverage in the poorest quintile increasing from nearly zero to just over 10%. In Pakistan, the level of coverage in all quintiles except the poorest showed improvement over time – with a consequent increase in the level of pro-rich inequality. In Viet Nam, coverage in the poorest quintile remained fairly stable while that in each of the other wealth quintiles – like the levels of pro-rich and pro-urban inequalities – initially increased but then declined.

## Discussion

Our findings indicate that, despite progress, much remains to be done if the benefit of routine childhood immunization is to be maximized. Reports of regional levels of vaccination coverage may mask local challenges, inequalities and variation. We observed pro-rich inequalities in full immunization coverage in most low- or middle-income countries, although they were, in general, relatively small in the Region of the Americas and European Region – and relatively large in the Eastern Mediterranean and Western Pacific Regions. Pro-urban inequalities were also common. They were generally very small in the low- or middle-income countries in the Region of the Americas and largest in such countries in the Eastern Mediterranean region, where coverage was about 60% higher among urban children than among rural children. Low- or middle-income countries in the South-East Asia Region showed the largest absolute pro-male inequalities.

We observed that, whether related to sex, wealth or urban/rural residence, inequalities in full immunization coverage varied substantially between and within our study countries. Inequalities related to wealth and urban/rural residence appeared to be ubiquitous and persistent and to be larger, in general, than the corresponding sex-related inequalities. Although some countries have made substantial progress in reducing such inequalities, some other countries have seen such disparities increase.

Among the eight countries included in our investigation of temporal trends in coverage and coverage inequality, Madagascar and Mozambique appeared to have made the most progress in improving national levels of coverage – in both cases by achieving particularly rapid increases in coverage in the poorest quintile. In general, the factors that are believed to have contributed to global improvements in immunization coverage include national multi-year planning, district-level planning and monitoring and the establishment of national budget lines funded via domestic and external resources for the strengthening of immunization services.^25^ Our observation of markedly different temporal trends in coverage and coverage inequality in eight countries needs to be followed up with case studies aimed at documenting the factors – within and beyond the health sector – that might explain such variation.

It is important to note that the data on coverage being reported here are solely based on survey information. As international agencies estimate vaccination coverage using a combination of data from surveys and data from health information systems, the coverage levels reported here will not necessarily be consistent with the estimates given in official documents produced by national governments and the United Nations. However, such estimates cannot be disaggregated by wealth quintile or place of residence and can rarely be used to determine the level of the full immunization coverage that we wished to investigate. To allow consistent and meaningful comparisons, we confined our investigation to vaccines that are available in almost all countries of the world. We ignored several new vaccines that are included in the national immunization programmes of a few of our study countries. Another limitation of the present study is that, when vaccination cards are not available, the information collected on child immunization in national surveys has to be based on the recall of mothers or other caregivers.

Our results indicate that the ultimate goal of the *Global vaccine action plan 2011–2020* – i.e. universal access to immunization^3^ – will only be achieved if the relevant health workers, policy-makers and stakeholders can: (i) develop and implement strategies for reaching those who are difficult to reach and for promoting the need for full immunization among those who have contact with health services for other interventions; (ii) expand vaccination programmes to include underserved groups; (iii) improve the quality of the monitoring of immunization coverage; (iv) use monitoring data to ameliorate programme performance; and (v) explore additional cross-sectoral strategies – particularly in those low- or middle-income countries with the worst inequalities in coverage. The improvements in coverage and equitable access to routine immunizations achieved by some Latin American countries may serve as useful examples.^26^

## References

[1] Fulfilling the health agenda for women and children: the 2014 report. Geneva: World Health Organization; 2014. Available from: http://www.countdown2015mnch.org/documents/2014Report/Countdown_to_2015-Fulfilling%20the%20Health_Agenda_for_Women_and_Children-The_2014_Report-Conference_Draft.pdf [cited 2016 Jul 3].

[2] Immunization summary. A statistical reference containing data through 2013. New York: United Nations Children’s Fund; 2014. Available from: http://www.who.int/immunization/monitoring_surveillance/Immunization_Summary_2013.pdf [cited 2016 Jul 3].

[3] Global vaccine action plan 2011–2020. Geneva: World Health Organization; 2013. Available from: http://www.who.int/iris/bitstream/10665/78141/1/9789241504980_eng.pdf [cited 2016 Jul 3].

[4] Jani JV, De Schacht C, Jani IV, Bjune G. Risk factors for incomplete vaccination and missed opportunity for immunization in rural Mozambique. BMC Public Health. 2008 5 16;8(1):161. 10.1186/1471-2458-8-161 18485194 PMC2405792

[5] Akmatov MK, Mikolajczyk RT. Timeliness of childhood vaccinations in 31 low and middle-income countries. J Epidemiol Community Health. 2012 7;66(7):e14. 10.1136/jech.2010.124651 21551179

[6] Pande RP, Yazbeck AS. What’s in a country average? Wealth, gender, and regional inequalities in immunization in India. Soc Sci Med. 2003 12;57(11):2075–88. 10.1016/S0277-9536(03)00085-6 14512239

[7] Pande RP. Selective gender differences in childhood nutrition and immunization in rural India: the role of siblings. Demography. 2003 8;40(3):395–418. 10.1353/dem.2003.0029 12962055

[8] Canavan ME, Sipsma HL, Kassie GM, Bradley EH. Correlates of complete childhood vaccination in East African countries. PLoS One. 2014 4 21;9(4):e95709. 10.1371/journal.pone.0095709 24752178 PMC3994083

[9] Antai D. Inequitable childhood immunization uptake in Nigeria: a multilevel analysis of individual and contextual determinants. BMC Infect Dis. 2009 11 20;9(1):181. 10.1186/1471-2334-9-181 19930573 PMC2787508

[10] Antai D. Faith and child survival: the role of religion in childhood immunization in Nigeria. J Biosoc Sci. 2009 1;41(1):57–76. 10.1017/S0021932008002861 18471339

[11] Gyimah SO. What has faith got to do with it? Religion and child survival in Ghana. J Biosoc Sci. 2007 11;39(6):923–37. 17359561 10.1017/S0021932007001927

[12] Mutua MK, Kimani-Murage E, Ettarh RR. Childhood vaccination in informal urban settlements in Nairobi, Kenya: who gets vaccinated? BMC Public Health. 2011 1 04;11(1):6. 10.1186/1471-2458-11-6 21205306 PMC3024932

[13] Mitchell S, Andersson N, Ansari NM, Omer K, Soberanis JL, Cockcroft A. Equity and vaccine uptake: a cross-sectional study of measles vaccination in Lasbela district, Pakistan. BMC Int Health Hum Rights. 2009 10 14;9 Suppl 1:S7. 10.1186/1472-698X-9-S1-S7 19828065 PMC3226239

[14] Requejo JH, Bryce J, Barros AJ, Berman P, Bhutta Z, Chopra M, et al. Countdown to 2015 and beyond: fulfilling the health agenda for women and children. Lancet. 2015 1 31;385(9966):466–76. 10.1016/S0140-6736(14)60925-9 24990815 PMC7613194

[15] Mhatre SL, Schryer-Roy AM. The fallacy of coverage: uncovering disparities to improve immunization rates through evidence. Results from the Canadian International Immunization Initiative Phase 2 - Operational Research Grants. BMC Int Health Hum Rights. 2009 10 14;9 Suppl 1:S1. 10.1186/1472-698X-9-S1-S1 19828053 PMC3226229

[16] Corsi DJ, Bassani DG, Kumar R, Awasthi S, Jotkar R, Kaur N, et al. Gender inequity and age-appropriate immunization coverage in India from 1992 to 2006. BMC Int Health Hum Rights. 2009 10 14;9 Suppl 1:S3. 10.1186/1472-698X-9-S1-S3 19828061 PMC3226235

[17] Clark A, Sanderson C. Timing of children’s vaccinations in 45 low-income and middle-income countries: an analysis of survey data. Lancet. 2009 5 2;373(9674):1543–9. 10.1016/S0140-6736(09)60317-2 19303633

[18] Data [Internet]. Rockville: DHS Program; 2015. Available from: http://dhsprogram.com/Data/ [cited 2015 Apr 12].

[19] Multiple Indicator Cluster Surveys [Internet]. New York: United Nations Children’s Fund; 2015. Available from: http://mics.unicef.org/ [cited 2015 Apr 12].

[20] Rutstein SO, Johnson K. The DHS wealth index. Calverton: ORC Macro; 2004.

[21] Barros AJ, Victora CG. Measuring coverage in MNCH: determining and interpreting inequalities in coverage of maternal, newborn, and child health interventions. PLoS Med. 2013;10(5):e1001390. 10.1371/journal.pmed.1001390 23667332 PMC3646214

[22] Harper S, Lynch J. Methods for measuring cancer disparities: using data relevant to healthy people 2010 cancer-related objectives. Bethesda (MD): National Cancer Institute; 2005.

[23] Wagstaff A, Paci P, van Doorslaer E. On the measurement of inequalities in health. Soc Sci Med. 1991;33(5):545–57. 10.1016/0277-9536(91)90212-U 1962226

[24] GAVI gender policy: frequently asked questions. Geneva: GAVI, The Vaccine Alliance; 2014. Available from: http://www.gavi.org/library/gavi-documents/policies/[cited 2015 Apr 12].

[25] Duclos P, Okwo-Bele JM, Gacic-Dobo M, Cherian T. Global immunization: status, progress, challenges and future. BMC Int Health Hum Rights. 2009 10 14;9 Suppl 1:S2. 10.1186/1472-698X-9-S1-S2 19828060 PMC2762311

[26] Restrepo-Méndez MC, Barros AJD, Requejo J, Durán P, Serpa LAF, França GVA, et al. Progress in reducing inequalities in reproductive, maternal, newborn, and child health in Latin America and the Caribbean: an unfinished agenda. Rev Panam Salud Publica. 2015;38(1):9–16. 26506316

